# FDG-PET/CT in a Patient with Poor-Risk Non-Seminoma Testis with Mature Teratoma and Secondary Gliosarcoma: Multimodality Imaging for Guiding Multimodality Treatment

**DOI:** 10.1007/s13139-015-0321-9

**Published:** 2015-02-13

**Authors:** Elske Quak, Iringo Kovacs, Wim J. G. Oyen, Winette T. A. van der Graaf

**Affiliations:** 1 Department of Nuclear Medicine, Radboud University Nijmegen Medical Centre, Nijmegen, The Netherlands; 2 Department of Pathology, Radboud University Nijmegen Medical Centre, Nijmegen, The Netherlands; 3 Department of Medical Oncology, Radboud University Nijmegen Medical Centre, Nijmegen, The Netherlands; 4 Department of Nuclear Medicine, François Baclesse Cancer Centre, Avenue Général Harris, 14076 Caen cedex 5, France

**Keywords:** FDG-PET/CT, Testicular cancer, Teratoma, Germ cell tumour

## Abstract

The value of F-18-fluorodeoxyglucose positron emission tomography/computed tomography (FDG-PET/CT) in detecting viable tumours in patients with metastasised non-seminomatous testicular cancer and residual and new masses post chemotherapy remains to be determined. We describe the case of a 41-year-old patient with metastasised non-seminomatous testicular cancer, with both retroperitoneal and extra-retroperitoneal residual masses post chemotherapy, for whom FDG-PET/CT guided major treatment decisions. FDG-PET/CT correctly identified the locations of viable tumour, as was proved by histology, and successfully guided surgery. In conclusion, in selected cases surveillance of patients with non-seminomatous testicular cancer with FDG-PET/CT can guide major treatment decisions when considering surgery for metastatic disease.

## Introduction

The role of F-18-fluorodeoxyglucose positron emission tomography/computed tomography (FDG-PET/CT) to detect viable tumour in the follow-up of patients with non-seminoma testis and residual masses is still not conclusively shown, and studies to further define this role show variable results [1–5]. However, the value of FDG-PET/CT in the follow-up of individual patients with mature teratoma and secondary gliosarcoma can be essential in guiding the sequence and extent of further treatment, as illustrated by the following case.

## Case report

A 41-year-old man first visited the urologist in a general hospital because of gynaecomasty and a mass in the right groin 6 years ago. He had a history of a left-sided orchidectomy as a baby, and a surgical correction of a right-sided inguinal hernia at the age of 31. Because of an abnormal ultrasound of the right testicle and increased tumour markers in the blood, a radical inguinal right-sided funiculo-orchidectomy was performed, and a testicular prosthesis was placed. The resection specimen showed a non-seminomatous testicular cancer, which consisted purely of embryonic cell carcinoma without components of teratoma. The contrast-enhanced CT of the thorax and abdomen performed the day after the orchidectomy showed extensive abdominal lymph node metastases, multiple liver metastases, metastases in the left adrenal gland and metastases in the right inguinal region.

**Fig. 1:**
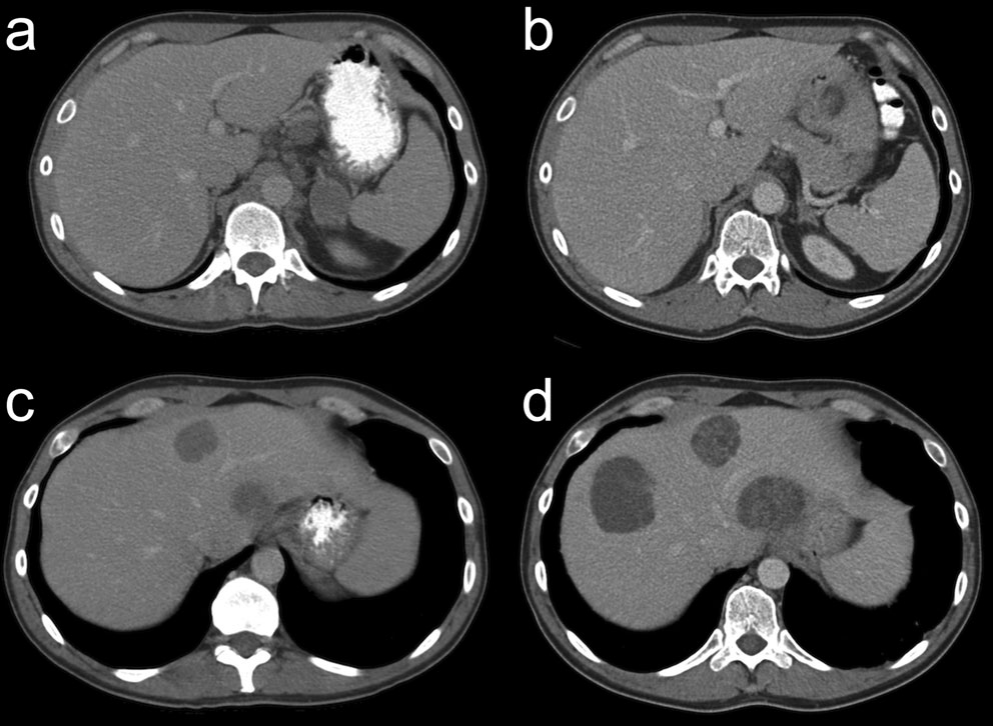
Contrast-enhanced axial CT images before (**a**, **c**) and after (**b**, **d**) the first line of chemotherapy showed a regression of the left adrenal metastases (**a**, **b**) and a clear progression of the liver metastases (**c**, **d**)

Because of this poor-risk non-seminomatous testicular cancer, the patient was referred to the Oncology Department of our University Medical Centre. Initial serum markers were: lactate dehydrogenase (LDH) 1,534 U/l (normal, <250 U/l), beta-human chorionic gonadotropin (beta-HCG) 17.5 ng/ml (normal, <1.0 ng/ml) and alpha-fetoprotein (AFP) 527 μg/l (normal, <3.0 μg/l). Chemotherapy with bleomycin, etoposide and cisplatin (BEP) was started immediately. The patient tolerated the four cycles of chemotherapy relatively well. Within 2 months after the start of chemotherapy, the serum tumour markers normalised. However, contrast-enhanced CT after completion of the chemotherapy showed a mixed response: the lesion in the left adrenal gland and the abdominal lymph node metastases had declined, but the lesions in the liver and the right inguinal region showed an increase in size (Fig. 1). Because of the normal serum tumour markers and the radiological aspects of these growing lesions, growing teratoma was suspected. Biopsy of one of the liver lesions showed no malignancy. Whilst planning extensive surgery for the suspected teratoma lesions in the liver, the patient unfortunately presented with a biochemical relapse (AFP, 47.1 μg/l) of his non-seminomatous testicular cancer and a deterioration of his renal function due to obstruction of the right ureter by a tumour mass. The surgery was postponed and a double-J catheter was inserted in the obstructed right ureter, after which the renal function normalised. To evaluate the extent of the disease, FDG-PET/CT was performed, which showed multiple small liver lesions with a high metabolic activity and two highly metabolic active regions in the right inguinal mass, all suspected to be metabolically active malignant tumour. FDG-PET/CT also showed two large liver lesions without increased FDG uptake, probably teratomas. Because of the recurrent non-seminomatous testicular cancer, the patient was treated with four cycles of second line chemotherapy consisting of paclitaxel, ifosfamide and cisplatin (TIP). The serum tumour markers normalised and follow-up FDG-PET/CT performed after two cycles of chemotherapy showed a complete metabolic response in all lesions.

**Fig. 2:**
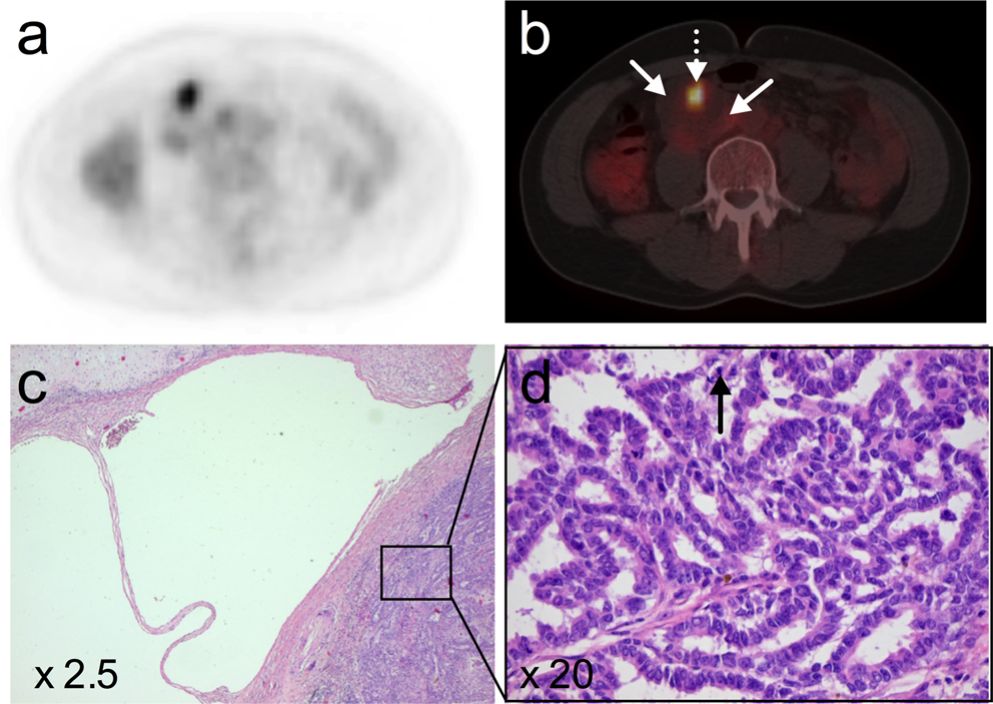
The FDG-PET (**a**) and FDG-PET/CT (**b**) images showed a hypermetabolic region (*dotted arrow*) in an otherwise relatively photopenic mass (*straight arrows*) in the retroperitoneum. Histology after surgical excision showed mature teratoma with an astrocytoma grade II component (not shown) and a papillary carcinoma component (**c**). Note the cystic spaces consistent with mature teratoma and the papillary carcinoma in the right lower corner of the image (**c**), with glandular and papillary structures, atypical cells and mitotic figures (**d**)

However, a further FDG-PET/CT performed after completion of the four cycles of chemotherapy showed new, highly metabolically active lymph nodes in the retro peritoneum again suggestive for viable tumour, whilst the serum tumour markers remained normal. The patient underwent a retroperitoneal lymph node dissection, including resection of these FDG avid regions, which showed mature teratoma with secondary malignant components of papillary carcinoma and astrocytoma grade II (Fig. 2).

**Fig. 3:**
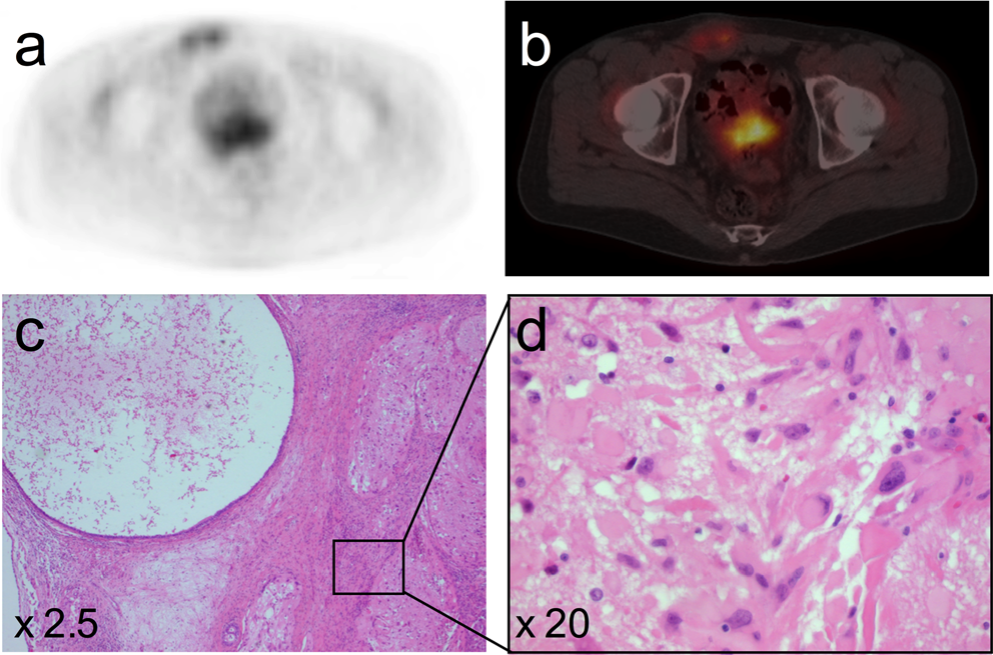
The FDG-PET (**a**) and FDG-PET/CT (**b**) images showed hypermetabolic areas in the right inguinal mass. Histology after the inguinal lymph node dissection showed cystic spaces consistent with mature teratoma and a neuroglial component with uncertain malignant potency (**c**, **d**). There were mitotic figures (not shown)

**Fig. 4:**
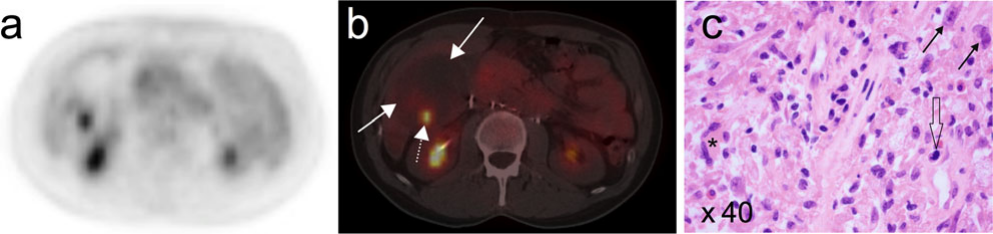
The FDG-PET (**a**) and FDG-PET/CT (**b**) images showed a hypermetabolic region (*dotted arrow*) in an otherwise photopenic teratoma (*straight arrows*) in the liver. Histology after the partial liver resection showed a partial secondary malignant dedifferentiation of the teratoma, possibly gliosarcoma or pleomorphic malignant fibrous histiocytoma (**c**). Note the atypical (*solid arrows*) and giant cells (*asterisk*) and the mitotic figure (*open arrow*)

A few months later, a follow-up FDG-PET/CT showed increasing metabolic activity in the right inguinal mass and in a liver lesion. First, a right sided inguinal lymph node dissection was done, which showed teratoma with a neuroglial component of uncertain malignant potency (Fig. 3). Subsequently, a partial resection of the liver was performed, which showed teratoma with the same known neuroglial component and a lesion with secondary malignant degeneration, possibly gliosarcoma or pleomorphic malignant fibrous histiocytoma (Fig. 4).

After surgery, the patient was in excellent health and remained free of disease for more than a year. However, during follow-up, FDG-PET/CT showed a hypermetabolic tumour in the musculature of the right upper leg. To rule out metastases, this lesion was excised, and it appeared to be a nodular fasciitis on pathological examination.

The first follow-up FDG-PET/CT, 2 months after resection of the nodular fasciitis, showed increasing metabolic activity in one of the teratomas in the liver and in the postsurgical region in the liver. Again, the affected segments were excised, which showed mature teratoma with secondary malignant degeneration with neuroglial and probably mesenchymal components.

At present, the patient is 6 years after his primary diagnosis of poor risk non-seminomatous testicular cancer with secondary somatic malignant dedifferentiated mature teratoma. The last surgery is now more than 3 years ago. The patient remains in an excellent clinical condition, is working full time, plays water polo and has no evidence of disease, neither biochemically nor on multimodality imaging with FDG-PET/CT.

## Discussion

This case clearly illustrates the potential of FDG-PET/CT in the individual patient with metastasised non-seminomatous testicular cancer and residual masses post chemotherapy, both in the retroperitoneum and extra-retroperitoneal. The risk that these masses contain viable germ cell tumour on pathological examination is reported to be about 10 % and is associated with poor clinical outcome [6]. We feel that the outcome of this patient has clearly benefitted from a multidisciplinary approach, leading to multiple successful FDG-PET/CT-guided surgical interventions.

The ability of FDG-PET to differentiate viable germ cell tumour from necrosis, fibrosis or teratoma in non-seminomatous testicular cancer patients with residual masses after chemotherapy was shown by several studies [7, 8]. The FDG uptake in viable tumour was significantly higher than the generally low FDG uptake in necrosis, fibrosis or teratoma. However, FDG-PET could not differentiate between teratoma, low-grade malignancy and fibrosis or necrosis. The clinical relevance of these findings is debatable, because in general, the consensus is that teratomas, hypermetabolic or not, should be removed because of the potential of malignant dedifferentiation and because growth can cause mechanical problems [9–11]. In this particular case, however, resection of all the teratomas in the liver would only be possible by performing a liver transplantation. FDG-PET/CT allowed for correct localisation of the malignant tissue and served as a guide for the surgical interventions.

The overall performance of FDG-PET compared with contrast-enhanced CT and serum tumour markers in patients with residual masses after chemotherapy in non-seminomatous testicular cancer was prospectively studied by Oechsle et al. [3], with histological confirmation in all patients. The accuracy of FDG-PET in the detection of viable tumour was not significantly better than that of CT or serum tumour markers (56, 55 and 56 % respectively). Sensitivities and specificities of FDG-PET for the detection of viable tumour reported in the literature vary between 59 and 70 % and 48 and 92 %, respectively [2, 3]. Hain et al. [12] reported a management change due to FDG-PET in 57 % of patients with testicular cancer. However, it must be noted that most of the FDG-PET studies in metastasised non-seminomatous testicular cancer, including these studies, were performed on earlier generation (stand-alone) PET scanners. Nowadays, hybrid PET/CT scanners are used, allowing for anatomical correlation in PET interpretation, and new techniques in image reconstruction, such as time-of-flight, improve spatial resolution in PET imaging. In our experience, these techniques improve the overall performance of FDG-PET/CT, and we expect the results of FDG-PET/CT in metastasised non-seminomatous testicular cancer to be superior to the aforementioned results, as is suggested in a recent study by Sterbis et al. [13] using fusion imaging in testicular cancer.

This patient also demonstrated a non-malignant hypermetabolic muscular lesion in the right upper leg, which proved to be a nodular fasciitis. Non-malignant, positive FDG-PET/CT findings due to inflammatory conditions, for example, are a well-known caveat in the differential diagnosis of FDG-accumulating lesions, as these can mimic or coexist with malignancy [14].

In conclusion, in selected cases, surveillance of patients with non-seminomatous testicular cancer with FDG-PET/CT can guide major treatment decisions when considering surgery for metastatic disease, mature teratoma or even secondary degenerated mature teratoma.
